# High-Density Polyethylene/Carbon Black Composites in Material Extrusion Additive Manufacturing: Conductivity, Thermal, Rheological, and Mechanical Responses

**DOI:** 10.3390/polym15244717

**Published:** 2023-12-15

**Authors:** Nectarios Vidakis, Markos Petousis, Nikolaos Michailidis, Nikolaos Mountakis, Apostolos Argyros, Mariza Spiridaki, Amalia Moutsopoulou, Vassilis Papadakis, Costas Charitidis

**Affiliations:** 1 Department of Mechanical Engineering, Hellenic Mediterranean University, 71410 Heraklion, Greece; vidakis@hmu.gr (N.V.); mountakis@hmu.gr (N.M.); mspyridaki@hmu.gr (M.S.); amalia@hmu.gr (A.M.); 2 Physical Metallurgy Laboratory, Mechanical Engineering Department, School of Engineering, Aristotle University of Thessaloniki, 54124 Thessaloniki, Greece; nmichail@auth.gr (N.M.); aargyros@auth.gr (A.A.); 3 Centre for Research & Development of Advanced Materials (CERDAM), Center for Interdisciplinary Research and Innovation, Balkan Centre, Building B’, 10th km Thessaloniki-Thermi Road, 57001 Thessaloniki, Greece; 4 Department of Industrial Design and Production Engineering, University of West Attica, 12244 Athens, Greece; v.papadakis@uniwa.gr; 5 Institute of Electronic Structure and Laser, Foundation for Research and Technology–Hellas, N. Plastira 100m, 70013 Heraklion, Greece; 6 Department of Materials Science and Engineering, School of Chemical Engineering NTUA, National Technical University, Iroon Polytechneiou 9, Zografou, 15780 Athens, Greece

**Keywords:** high-density polyethylene, carbon black, material extrusion, additive manufacturing, 3D printing, electrical conductivity, composites, mechanical properties, melt flow index

## Abstract

High-density polyethylene polymer (HDPE) and carbon black (CB) were utilized to create HDPE/CB composites with different filler concentrations (0.0, 2.0, 4.0, 6.0, 8.0, 10.0, 16.0, 20.0, and 24.0 wt.%). The composites were extruded into filaments, which were then utilized to fabricate 3D-printed specimens with the material extrusion (MEX) method, suitable for a variety of standard mechanical tests. The electrical conductivity was investigated. Furthermore, thermogravimetric analysis and differential scanning calorimetry were carried out for all the HDPE/CB composites and pure HDPE. Scanning electron microscopy in different magnifications was performed on the specimens’ fracture and side surfaces to investigate the morphological characteristics. Rheological tests and Raman spectroscopy were also performed. Eleven different tests in total were performed to fully characterize the composites and reveal connections between their various properties. HDPE/CB 20.0 wt.% showed the greatest reinforcement results in relation to pure HDPE. Such composites are novel in the MEX 3D printing method. The addition of the CB filler greatly enhanced the performance of the popular HDPE polymer, expanding its applications.

## 1. Introduction

Additive manufacturing (AM) is a technology constantly gaining popularity [1,2]. Fused filament fabrication (FFF) is one of the methods of additive manufacturing, belonging to the material extrusion (MEX) family of technologies [3,4]. This 3D printing technique is often used to contribute to applications related to construction, apparel, dentistry, and medicine applications and industries related to electronics, robotics, aerospace, as well as automotive [5,6]. It is useful for producing especially lightweight parts for a variety of industries, such as the aerospace industry [7,8,9,10,11]. It allows for the 3D printing of tissues and implants in biomedicine [12,13]. However, the parameters utilized in FFF must be carefully selected and managed, since they significantly affect the 3D-printed components’ dimensional accuracy (DA) and surface quality (SQ) [14]. It should be noted that FFF is considered to be a technique of low material waste (a sustainable technique) and is suitable for developing multifunctional materials [15,16].

Materials are essential in the development of innovative machinery and parts for a variety of industries [17,18]. The concept of integrating materials arose since no material could satisfy all product requirements [19,20]. Therefore, composite materials combine two or more constituents [21,22]. There are numerous methods for strengthening the link between the polymer matrix and reinforcing additives, such as fibers, for example, natural fibers [23], glass fibers [24], or recyclable fibers [25]. Composites have quickly expanded their use due to their distinctive and improved properties [26,27]. Specifically, polymer composites offer a variety of qualities [28]. To determine how 3D printing affects the material’s structural properties, the mechanical performance of extruded composite filaments is also examined [29,30,31]. The most frequently generated filaments in the production of composites are aramid, glass, and carbon [32]. A growing field of research is the use of carbon-reinforced composite materials in additive manufacturing [33,34]. Carbon-based nanomaterials are considered lightweight and can provide the composite with high strength and versatility [35,36,37]. Numerous industrial and domestic applications, including self-regulating heating elements, fluid sensors, current switches, capacitors, thermal controllers, and electromagnetic interference shielding, make use of these CB-reinforced composites [38].

The vast majority of plastic components are produced all over the world using polymer resins like low-density polyethylene (LDPE), high-density polyethylene (HDPE), polystyrene (PS), and polyethylene terephthalate (PET) [39]. As a thermoplastic material, it is frequently used in films, wire insulation, containers, sheets, pipelines, and cables [40]. The use of electrically conductive polymer composites containing carbon black (CB), in electronic packaging and electromagnetic interference (EMI) shielding applications appears promising. Low density, long-lasting conductivity, cost-effectiveness, and chemical stability are only a few advantages of CB [41]. As reported in the literature, the mechanical properties of the polymer lattice, such as tensile and flexural strength, are improved by adding carbon black. However, the composite’s impact strength tends to deteriorate over time [42,43]. Mechanical property variations are caused by variations in morphology, filler density, geometrical features, and the matrix and filler interface adhesion [44]. Additionally, carbon-based fillers, such as single-wall carbon nanotubes (SWCNT) [45,46], graphene [47], diamond-like carbon (DLC) [48,49], and carbon black doped with nano silver [50], exhibit thermoelectric and antimicrobial properties, according to the existing literature [51].

Liu and Horrocks examined how a carbon black masterbatch affected the mechanical and UV resistance qualities of a linear low-density polyethylene (LLDPE) film’s surface (of 75 μm in thickness) after being exposed to artificial weathering. The findings showed that the blended films’ UV resistance strength increased when carbon black concentration rose from 1.5 to 3.5 wt.% [52]. Parvin et al. examined the effects of independently adding inorganic talc and organic carbon black fillers to high-density polyethylene (HDPE). Samples were created for morphological and mechanical property evaluation using a compression molding machine at 160 °C. According to the study, HDPE performs better with carbon black than talc [53]. Liang and Yang [54,55] recently investigated the HDPE/CB composites’ electrical conductivity properties. In-depth research was completed on topics such as resistivity relaxation behavior [54], the impact of heat treatment on conductivity [56], and the effect of carbon fiber content and scale on electrical conductivity in HDPE/CF structures [57]. According to their research, resistivity relaxation occurred in an exponential pattern at temperatures higher than the melting point of the resin. In [58], thermal black (TB), was utilized as a filler to create composites with PP (polypropylene), PA6 (polyamide 6), PPS (polyphenylene sulfide), and ABS (acrylonitrile butadiene styrene). The filler percentage ranged between 1 and 40 wt.%. Filaments were created and then 3D printed to investigate their mechanical, thermal, morphological, and rheological properties, similar to the study herein. The results showed that there were many advantages provided by the addition of TB in thermoplastic polymers and the total amount of the compounds showed electrical resistance. In [59], CB was used as the filler of epoxy resin at 0.5, 1.0, and 2.0 wt.% concentrations (quantities lower than the study herein), and then samples were fabricated with silicon molds. Compared to pure PA12, 0.5 wt.% showed less fracture toughness, 1.0 wt.% showed almost similar fracture toughness, and 2.0 wt.% revealed higher fracture toughness. Additionally, there is an investigation on ABS/CB composites, which also underwent electrical conductivity testing, and it was proved that although the rise of CB percentage increases the electrical conductivity (which agrees with the study herein) the mechanical properties are reduced (which does not match the results of this study) [60].

Herein, new and more robust composites are being developed and explored by adding carbon black to an HDPE matrix at predetermined weight ratios. Composite filaments are created during this investigation using the melt extrusion technique. Due to the addition of carbon black particles, the results of 3D-printed samples show an increase in both tensile and flexural strength. The electrical properties were also significantly improved. The study also utilizes methods such as Raman spectroscopy and scanning electron microscopy (SEM). Furthermore, thermogravimetric analysis (TGA) and differential scanning calorimetry (DSC) are used to evaluate the heat-related properties of the manufactured materials. In this extensive effort, the authors wanted to fully understand the response of the prepared composites, find potential weaknesses, and attempt to find connections between the different properties studied. The research contributed to better comprehending the potential advantages and restrictions of this material combination in various practical applications by filling research gaps. The use of HDPE combined with additives such as carbon black in 3D printing may be motivated by the goal of improving mechanical qualities, UV resistance, and electrical conductivity while obtaining a budget-friendly solution.

## 2. Materials and Methods

**Figure 1:**
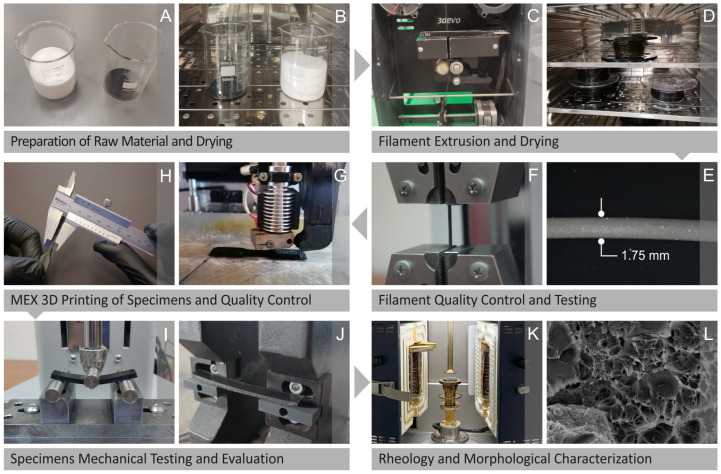
Presentation of the procedure followed in the research, namely, the (**A**) preparation and (**B**) drying of the raw material, (**C**) extrusion and (**D**) drying, (**E**) quality control and (**F**) testing of the filament, (**G**) MEX 3D-P fabrication and (**H**) quality control, (**I**) mechanical testing, (**J**) evaluation, and (**K**) rheological and (**L**) morphological analysis of the specimens.

Figure 1 presents the procedures of the research, namely, the preparation (Figure 1A) and drying (Figure 1B) of the raw material, the extrusion (Figure 1C), drying (Figure 1D), quality control (Figure 1E), and testing (Figure 1F) of the filament. It also shows the MEX 3D printing (3D-P) process (Figure 1G), quality control (Figure 1H), mechanical testing (Figure 1I), and evaluation (Figure 1J) of the specimens as well as the rheological (Figure 1K) and morphological (Figure 1L) characterization.

### 2.1. Materials

**Figure 2:**
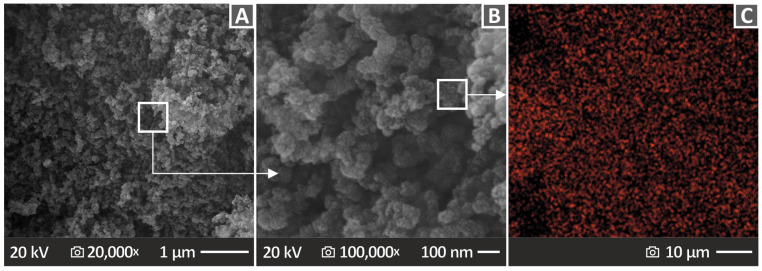
SEM analysis of CB raw material at (**A**) 20,000× and (**B**) 100,000× magnification; (**C**) EDS mapping image of the carbon element taken from the CB raw material.

Herein, Kritilen HDPE, provided in a powder form of industrial grade, was utilized as the matrix material of the study. The HDPE powder was sourced from Plastika Kritis S.A. (Heraklion, Greece). The value of density, according to the information provided by the supplier, was 0.960 g/cm^3^, 7.5 g/10 min was the mass-flow rate, and 127 °C was the Vicat softening temperature. CB, specifically the C-NERGY Super P Conductive Carbon Black grade, originating from Nanografi Nanotechnology AS, Ankara, Turkey, was utilized as the reinforcing material. Figure 2 shows SEM images captured from the raw material of CB at 20,000× magnification (Figure 2A) and 100,000× magnification (Figure 2B), as well as the Energy-dispersive X-ray spectroscopy (EDS) mapping image of the carbon element taken on the CB (Figure 2C). The nanoscale size of the particles was verified, and their shape is presented. The EDS mapping image verifies the presence and the uniform distribution of the carbon element in the nanopowder procured by Nanografi Nanotechnology AS.

### 2.2. Fabrication of Filament and Specimens

Both HDPE and CB raw materials were dried in the oven at 60 °C temperature overnight, and then separate mixtures, consisting of different filler loadings, were created. The prepared composites were HDPE/2.0 wt.% CB, HDPE/4.0 wt.% CB, HDPE/6.0 wt.% CB, HDPE/8.0 wt.% CB, HDPE/10.0 wt.% CB, HDPE/16.0 wt.% CB, HDPE/20.0 wt.% CB, and HDPE/24.0 wt.% CB. In the study, the CB loading in the composites was initially set to 2 wt.%. Lower filler loading was not expected to have an impact on the electrical conductivity of the samples [61,62]. This was verified by the electrical conductivity measurements performed afterward. Specimens were prepared, with the MEX 3D printing method, with the composite having the initial CB loading of 2 wt.%. These specimens were tested in all the tests carried out in the study. Then, a powder mixture with the CB weight percentage increased to 4 wt.% was extruded into a filament. This filament was utilized to manufacture specimens with composites with a 4 wt.% CB content. Again, tests were carried out on these specimens. This process of gradually increasing the CB content in the composites and testing the prepared samples was continued until the mechanical performance of the samples reached a high peak and then started to decrease. Up to 20 wt.% CB in the composites, as shown below, the mechanical performance increased in the composites. At the 24 wt.% samples, the mechanical performance of the samples drastically dropped. This was an indication that saturation of the CB filler in the HDPE matrix occurred, which led to inferior mechanical properties in the samples (in our case, both the tensile and the flexural strength were reduced, as presented below). That is why composites with CB content up to 24 wt.% were prepared and tested in the research, which is a rather high concentration for a filler with particles in the nanoscale. Still, the exact saturation threshold for the CB filler in the HDPE matrix, with the thermomechanical preparation process followed, was not determined, as it was not within the scope of the research.

For the preparation of the composites, the mixing procedure was carried out by using a laboratory mixer of high power. Along with the HDPE/CB composites, pure HDPE was also prepared and tested as a control sample, for its performance to be compared with the performance resulting from the reinforcement of CB with HDPE. A common issue when preparing composites is the dispersion of the particles in the matrix. To achieve the best possible dispersion of the particles in the matrix, the composites were extruded two successive times, with a process analytically presented in a previous study [63]. In [63], the process was proven to be effective. Briefly, a Noztek Pro (Noztek, Shoreham-by-Sea, UK) desktop single-screw extruder was used to initially turn the mixed powders into filament. This filament was shredded into pellets (3D Evo Shredder, 3D Evo B.V., Utrecht, The Netherlands). Then, a 3D Evo Composer 450 (3D Evo B.V., Utrecht, The Netherlands) was used to produce the final filament compatible with the MEX 3D printing process (1.75 mm in diameter), which was used to manufacture the specimens. This extruder was used in the second extruder step, as it features a screw with specially designed geometry for mixing materials, according to its manufacturer.

The extrusion and 3D printing settings were determined with preliminary tests and by consulting the corresponding literature on the MEX 3D printing of HDPE [3]. As to the extruding settings in the final extrusion step (3D Evo Composer 450, 3D Evo B.V., Utrecht, The Netherlands), they were set to be identical for all of the nano-compounds of this study, and they were as follows: 190 °C temperature of zone 1 (close to the nozzle), 200 °C temperature of zone 2, 200 °C temperature of zone 3, and 190 °C temperature of the zone 4 (close to the hopper of the extruder). The rotational speed of the extruder’s screw was 6 rpm, the speed of the cooling fans located after the nozzle was at 85%, and the average value of the filament’s diameter was 1.75 mm, according to the recording results of the built-in sensor placed in the extruder. Filaments were dried before they were about to be fed to the 3D printer for the fabrication of the specimens for each test.

**Figure 3:**
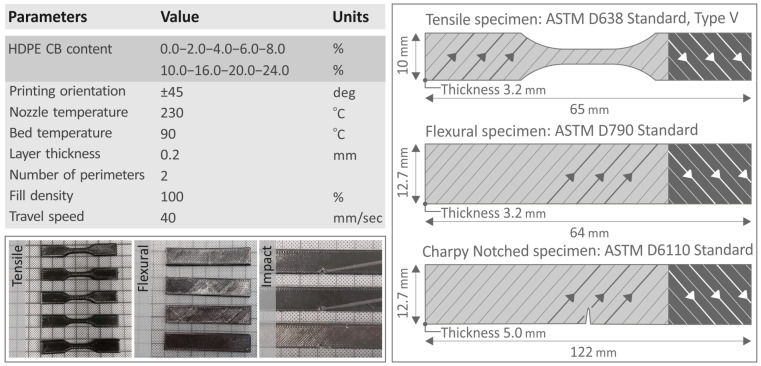
The 3D printing parameters for the fabrication of specimens and the fabricated specimens (left side of the figure), design, and dimensions of tensile, flexural, and Charpy notched specimens (right side of the figure), along with the corresponding standards. The arrows in the specimens indicate the orientation of the infill pattern used for the construction of the parts. The orientation is rotated 90 deg between the successive layers, aiming to reduce the anisotropy in the 3D-printed parts.

Pure HDPE and HDPE/CB specimens were designed with the Autodesk^®^ Fusion 360™ v. 16 (Autodesk^®^, Inc., San Francisco, CA, USA) software platform. Afterward, the created files were converted into Standard Tessellation Language (STL) format and then imported to the Intamsuite software v. 4.2 (Intamsys, Shanghai, China) for the creation of the G-code file. The specimens were manufactured through FFF 3D-P technology, by employing an Intamsys Funmat HT FFF 3D printer (Intamsys, Shanghai, China), with an all-metal 3D-P head. The printing parameters are shown in Figure 3 and were as follows: ±45 deg printing orientation, 230 °C nozzle temperature, 90 °C bed temperature, 0.2 mm layer thickness, 2 perimeters, 100% infill density, and 40 mm/sec travel speed. Regarding the infill pattern, a line pattern was used, and the built orientation of the line pattern was rotated 90 deg between successive layers, hence the ±45 deg printing orientation mentioned above. This reduces the anisotropy the 3D-printed parts have. For this reason, it was selected to be used in the 3D printing structure of the parts. The infill pattern and its change in the orientation are indicated on the right part of Figure 3 with respective arrows, inside the specimens’ geometry.

Figure 3 also presents the tensile, flexural, and impact specimens that were fabricated, with their dimensions and the standards on which they were based. It should be noted that the same extrusion and 3D printing settings were used in the composites and the control samples to attain comparable results. These settings were optimized for the control sample. Further optimization would be required for each composite. This would have achieved even more improved results. Still, the results between the composites and the control sample would not be comparable in this case. So, in the study, the same settings for all composites and pure HDPE were selected throughout the process.

### 2.3. Characterization Techniques

Both pure HDPE and HDPE/CB composites underwent Raman spectroscopy, by utilizing a scientific micro-Raman system named Labram HR-Horiba (Kyoto, Japan). It contains a laser module at 532 nm, with 90 mW of power to irradiate samples. Imaging was at the microscopic level and was performed with a 50× Olympus objective lens (LMPlanFL N, Tokyo, Japan) that has a 0.5 Numerical Aperture (NA) and a working distance of 10.6 mm. The laser power that reached the sample had 2 mW of power, by installing a 5% Neutral Density filter in the optical path. The Raman spectral resolution was around 2 cm^−1^. The imaging resolution was 1.7 μm laterally and 2 μm axially. The measurement spectral range was set from 50 up to 3900 cm^−1^. The exposure time per point was 10 s and, at each point, we made 5 accumulations. All areas after irradiation were inspected visually through the microscope to ensure no discoloration or degradation.

Thermogravimetric analysis was carried out under an oxygen atmosphere, on both pure HDPE and HDPE/CB composites 3D-P samples. The apparatus used for the measurements was a Perkin Elmer Diamond TG/TDA (Waltham, MA, USA), with a heating rate of 10 °C and a heating range between 30 °C and 550 °C. Additionally, a DSC analysis was carried out on a TA Instruments model 25 device (TA Instruments, New Castle, DE, USA). DSC was implemented in an inert atmosphere (nitrogen, N_2_). The heating scenario was implemented with a heating rate of 15 °C/min between 25 and 230 °C.

Additionally, rheology tests were carried out by utilizing a DHR/20 (Discovery-Hybrid-Rotational-Rheometer, also by TA-Instruments, New Castle, DE, USA) by utilizing the two parallel plates geometry along with the environmental conditions chamber to control the temperature to evaluate the rheological features of the nanocomposites. To avoid overheating and failure of the samples, an overall 10 s of data were captured for every point of measurement. To evaluate the flow characteristics of the composites via a hole with a predefined diameter and length under certain pressure and temperature conditions, melt flow rate (MFR) measures were carried out. The experimental parameters applied during the MFR measurements were in agreement with the international standard for MFR testing ASTM D1238-13 [64].

Scanning electron microscopy (SEM) was performed on the specimens in order to investigate their fracture and side surface. The apparatus employed was a field emission microscope (JSM IT700HR by Jeol Ltd., Tokyo, Japan), which was in high vacuum mode and had a 20 kV acceleration voltage. Moreover, the specimens were sputtered with gold during image capturing, in order to prevent them from charging.

HDPE/CB composite filaments of 1.75 mm diameter samples were tested for their electrical conductivity. The device utilized for the measurements was an Agilent Multimeter (Agilent 34401A6 ½, Agilent, Santa Clara, CA, USA). Thermal images were taken with a Flir One Pro thermal imaging camera (Teledyne Flir, Wilsonville, OR, USA). For the electrical conductivity measurements, random sections of the 1.75 mm in diameter filament were measured. The length of the samples was approximately 50 mm. In the middle of a section of l=30 mm in length, a mask was applied to prevent this area from being covered with the electroconductive paste, which was applied at the ends of the filament sample. A voltage was applied at the ends of the sample. Initially, a 0.1 Volt was applied, and the electric current (amperage) was measured. The voltage was then increased, and measurement of the electric current (amperage) was again taken. The step of the increase was 0.1 Volt. This procedure was repeated until the filament caught fire. In each step of the process, the temperature of the filament was also recorded to assess the Joule heating effect on the filament. For each voltage, the resistance R (Ohm) of the filament was calculated, using the Ohms law equation:

(1) R=V/I

A graph of the voltage vs. the current was also formed, and the slope of the linear segment of the curve is also the resistance R of the filament. The surface area of the filament can be derived by the following equation since the filament has a circular cross-section:

(2) $$A=\frac{\pi \cdot d^{2}}{4}$$

where d is the filament diameter (1.75 mm).

The Siemens (S, 1/Ohm) was calculated by the following equation [65]:

(3) S=1/R

And the resistivity (ρ) was calculated by the following equation [65]:

(4) $$\rho =R\frac{A}{l}$$

The electrical conductivity (σ, S/m) was calculated by the following equation [65]:

(5) $$\sigma =\frac{1}{\rho }=length/(R\times Area)=S\times length/Area$$

A graph of the electrical conductivity vs. the CB content on the nanocomposites was then formed to show how the electrical conductivity changes with the increase in the CB content in the nanocomposites.

Tensile tests were carried out on the corresponding specimens with the assistance of an Imada MX2 (Imada Inc., Northbrook, IL, USA) device with standardized grips at 10mm/min elongation speed, according to the ASTM D638-02a standard [66]. The number of tested specimens was six of each fabricated material. Type V specimens with 3.2 mm thickness were tested at room temperature. The same device as the tensile tests was utilized for the flexural tests of the corresponding specimens, according to the ASTM D790-10 [67], but this time equipped with a three-point bending test setup. The tests were carried out on six specimens of each prepared composite with dimensions of 12.7 mm width, 3.2 mm thickness, and 64 mm length, at room temperature, with a 10 mm/min testing speed. A Terco MT 220 (Terco I & S AB, Kungens Kurva, Sweden) Charpy impact apparatus was used for the conduction of impact tests, of the corresponding specimens according to the ASTM D6110-04 international standard [68]. There were six notched specimens of each prepared composite, with dimensions of 12.7 mm width, 5.0 mm thickness, and 122 mm length. The conditions during the testing were constant and the hammer’s release height was 367 mm. An apparatus named Innova Test 300- Vickers (Innovatest Europe BV, Maastricht, The Netherlands) was utilized for the microhardness measurements according to the ASTM E384-17 international standard [69]. A total of 100 gF of force was applied, with a 10 s indentation duration, on six specimens of each fabricated material.

## 3. Results

### 3.1. Raman Results of HDPE/CB Composites and CB

**Figure 4:**
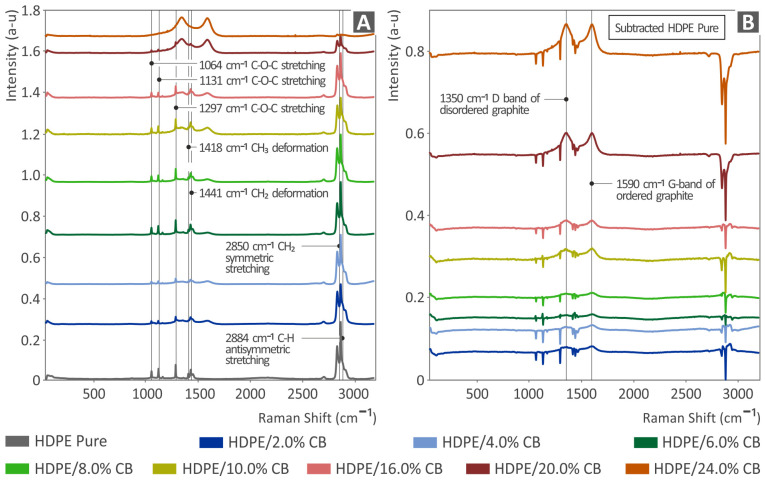
Results from (**A**) the Raman analysis carried out on pure HDPE and HDPE/CB composites as well as (**B**) the subtraction of pure HDPE from all HDPE/CB composites.

Figure 4A presents the Raman results of pure HDPE and HDPE/CB composites in all quantities, namely, 2.0, 4.0, 6.0, 8.0, 10.0, 16.0, 20.0, and 24.0 wt.%. Figure 4B presents the results from the subtraction of pure HDPE from the respective HDPE/CB composites. The Raman peaks from the pure HDPE sample were identified and compared with the literature and are presented in Table 1.

As presented in Figure 4A, the Raman peaks in most of the samples were derived from pure HDPE. We can see that C-O-C stretching was found at 1064, 1131, and 1297 cm^−1^. The CH_3_ and CH_2_ deformations were found at 1418 and 1441 cm^−1^, respectively. Moreover, CH_2_ symmetric stretching was found at 2850 cm^−1^ and C-H antisymmetric stretching at 2883 cm^−1^.

The addition of carbon black presented only the two peaks, one at 1350 cm^−1^, which is the graphite D-band of the disordered graphite, and the second at 1590 cm^−1^, which is the G-band of ordered graphite. Those large peaks grew as the concentration of CB was increased, until 20 wt.%, where they covered any Raman peaks from HDPE.

### 3.2. Thermogravimetric and Differential Scanning Calorimetry Analysis

**Figure 5:**
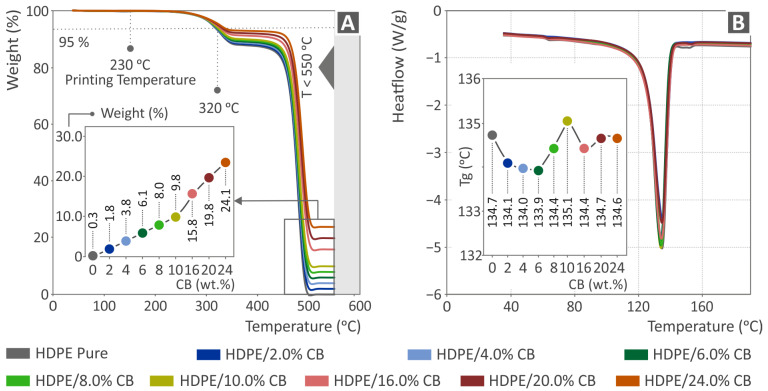
(**A**) TGA weight versus temperature and (**B**) DSC heat flow versus temperature graphs, regarding pure HDPE and HDPE/CB composites (the endothermic peak is presented).

Figure 5A presents the weight vs. temperature graph (acquired by the TGA) of pure HDPE as well as the HDPE/CB composites prepared for the study, at all filler loadings. Degradation of the material begins when the temperature reaches the value of approximately 320 °C, without important weight loss. It is observed that, as the filler percentage increases, the weight loss decreases, as expected. The addition of CB slightly increases the temperature at which the degradation occurs, but the difference is not important. The residual mass verified the filler loading in each composite. In addition, the temperatures used for filament extrusion and samples’ 3D printing are much lower than the acute degradation temperature of the composites. This ensures that no degradation of the materials occurred during the experimental process, which would have affected the acquired results.

On the other hand, the graph of heat flow vs. temperature (DSC), regarding pure HDPE as well as the HDPE/CB nanocomposites prepared for the study at all filler loadings, is presented in Figure 5B. There, it can be observed that the highest Tg value appears in the case of HDPE/10.0 wt.% CB. No linear relationship between the filler loading and the Tg temperature, or the differentiation of the heat flow rate, can be observed in the graph, and the effects of the filler in the base material are minor.

### 3.3. Viscosity and Melt Flow Rate Analyses

**Figure 6:**
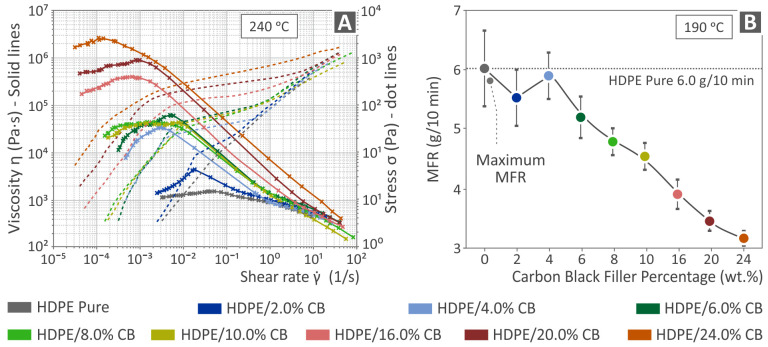
(**A**) viscosity and stress versus shear rate graph at 240 °C and (**B**) melt flow rate versus CB filler percentage, regarding pure HDPE and HDPE/CB composites.

Figure 6A presents the viscosity and stress vs. the shear rate graph at 240 °C, while Figure 6B shows the MFR (melt flow rate) vs. filler percentage graph, for pure HDPE and HDPE/CB nanocomposites. All the samples showed a shear-thinning, non-Newtonian, or pseudoplastic tendency, indicated by the overall drop in viscosity as the shear rate increased, which is a favorable material property for materials used in 3D printing applications. It is also obvious from Figure 6A that the higher the amount of the filler, the higher the viscosity and the stresses, while Figure 6B shows that the MFR in grams per 10 min decreases as the percentage of filler increases and also that the maximum MFR is found in the case of pure HDPE.

### 3.4. Mechanical Tests

**Figure 7:**
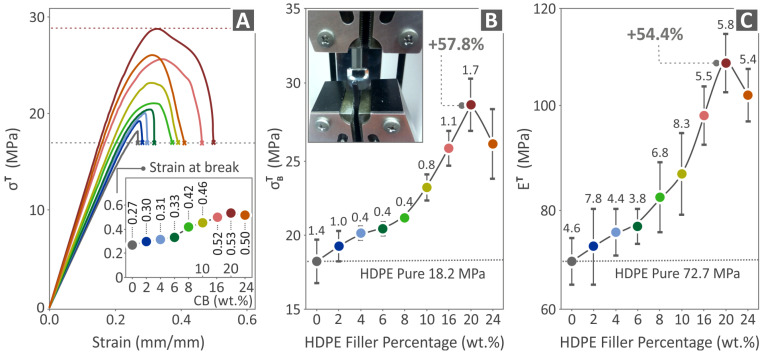
Results from the tensile tests carried out on the fabricated tensile specimens of pure HDPE and HDPE/CB composites: (**A**) tensile stress-to-strain graph (the inset graph shows the elongation at break for each nano-compound), (**B**) tensile strength, and (**C**) tensile modulus of elasticity.

Figure 7 shows the results of the tensile tests that the pure HDPE and all the HDPE/CB composites underwent. Figure 7A presents the tensile stress-to-strain graph of all composites, along with an image captured during the tensile testing of a random specimen. The inset graph of Figure 7A shows the elongation at break for each nano-compound. As shown, the elongation at break gradually (but not linearly) increases with the increase in the CB content in the samples up to 20 wt.%, and then it is slightly reduced. The highest value of the tensile stress is found in the case of HDPE/20.0 wt.% CB. Figure 7B shows the results regarding the tensile strength, where it is pointed out that the highest value is found in the case of HDPE/20.0 wt.% CB, 57.8% above pure HDPE. Figure 7C presents the results regarding the tensile modulus of elasticity, where the highest value of 54.4% above the pure HDPE is in the case of the HDPE/20.0 wt.% CB as well.

**Figure 8:**
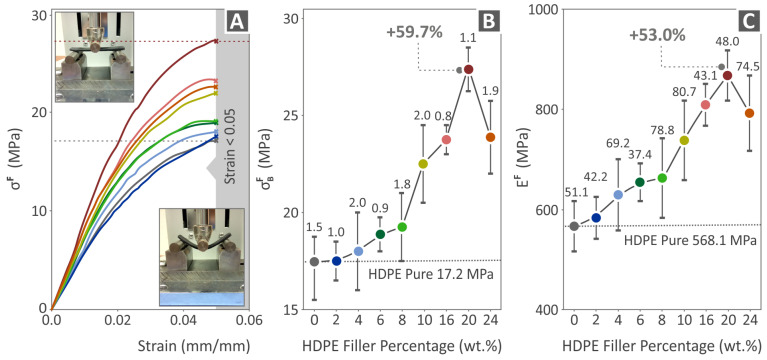
Results from the flexural tests carried out on the fabricated flexural specimens of pure HDPE and HDPE/CB composites: (**A**) graph of flexural stress to strain, (**B**) flexural strength, and (**C**) flexural modulus of elasticity.

Figure 8 shows the results of the pure HDPE, HDPE/2.0 wt.% CB, HDPE/4.0 wt.% CB, HDPE/6.0 wt.% CB, HDPE/8.0 wt.% CB, HDPE/10.0 wt.% CB, HDPE/16.0 wt.% CB, HDPE/20.0 wt.% CB, and HDPE/24.0 wt.% CB, regarding flexural tests. Figure 8A shows the flexural stress-to-strain graph of all composites as well as two images captured before and after the flexural testing of a random specimen. The highest value of flexural stress was found in the case of HDPE/20.0 wt.% CB. Figure 8B shows the flexural strength results, where the highest value 59.7% above pure HDPE is found in the case of HDPE/20.0 wt.% CB. Figure 8C contains results as to the flexural modulus of elasticity, where the highest value above pure HDPE is found in the case of HDPE/20.0 wt.% CB (53.0% higher than pure HDPE). It should be noted that the experiment was terminated at 5%, as no failure of the samples occurred at this point, following the instructions of the ASTM D790 standard.

**Figure 9:**
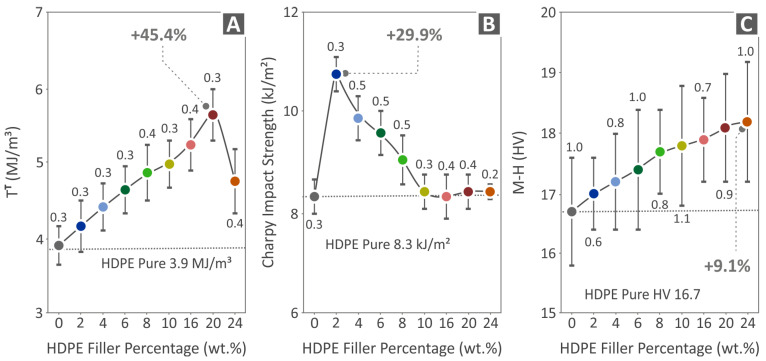
Results from the mechanical test carried out on the fabricated specimens, regarding the (**A**) tensile toughness, (**B**) Charpy impact strength, and (**C**) microhardness of pure HDPE and HDPE/CB composites.

Figure 9A shows the pure HDPE and HDPE/CB composites’ tensile toughness results, where the HDPE/20.0 wt.% CB value is 45.4% higher than the value of pure HDPE. Figure 9B presents the Charpy impact strength results of all composites, where HDPE/2.0 wt.% shows the highest value at 29.9% higher than pure HDPE. Figure 9C shows the microhardness results, where HDPE/24.0 wt.% CB presents the highest value at 9.1% above pure HDPE. It should be noted that, as shown in Figure 9C, the average microhardness is increasing, while the deviation is rather similar between the different nanocomposites. No high variations in the deviation are reported. The deviation is about 1 HV for all filler percentages, while the average microhardness values start from about 16.8 HV for the pure HDPE. Therefore, the deviation is less than 6% in the values, which is within the acceptable limits.

### 3.5. HDPE/CB Electrical Conductivity Presentation

**Figure 10:**
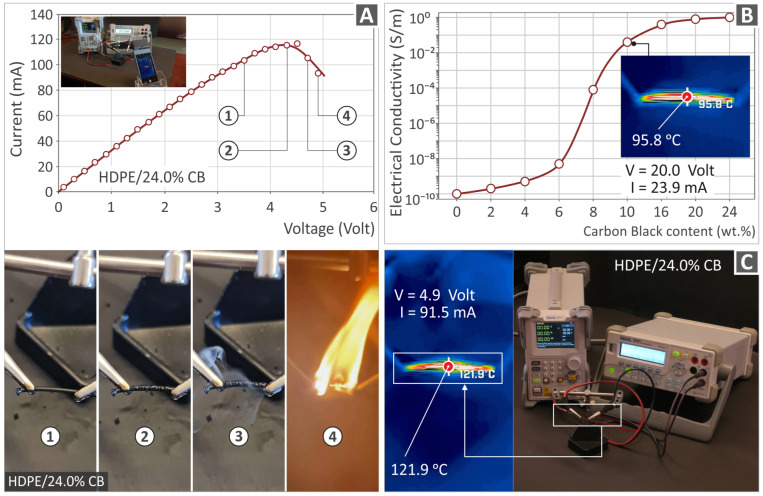
(**A**) Current versus voltage graph, regarding the HDPE/24.0 wt.% CB (top side of the figure), and a depiction of the filament during the respective different stages (bottom side of the figure); (**B**) electrical conductivity versus CB filler loading graph; (**C**) thermal image from the measurement of electrical conductivity at specific values of V (Volt) and I (mA).

The top side of Figure 10A shows the current vs. the voltage graph of HDPE/24.0 wt.% CB, along with an image captured during the measuring process. The bottom side of Figure 10A shows images of the filament in four different stages, which were captured during the process. As the voltage increased, the filament started to be heated, and it melted, with a visible change in its outer surface morphology (from smooth to rough, second picture of Figure 10A). With the further increase in the voltage (third picture of Figure 10A), the filament started to develop fumes, before it caught fire (fourth picture of Figure 10A), which showed that a limit in the current increase was reached.

Figure 10B presents the curve of electrical conductivity as to all of the CB percentages of filler. From the HDPE/6.0 wt.% CB and above, there is a drastic increase in the electrical conductivity, while it can be observed that the line created by the results of all composites, is sigmoidal-shaped, as it usually occurs from the polymer matrices combined with conductive fillers [75]. A digital IR-T (infrared thermography) image of the HDPE/10.0 wt.% CB filament is also included in Figure 10B. Figure 10C presents the joule heating electrothermal performance of the HDPE/24.0 wt.% CB composite, as well as a digital IR-T (infrared thermography) image of the HDPE/24.0 wt.% CB filament.

### 3.6. Analysis of Specimens through Scanning Electron Microscopy

**Figure 11:**
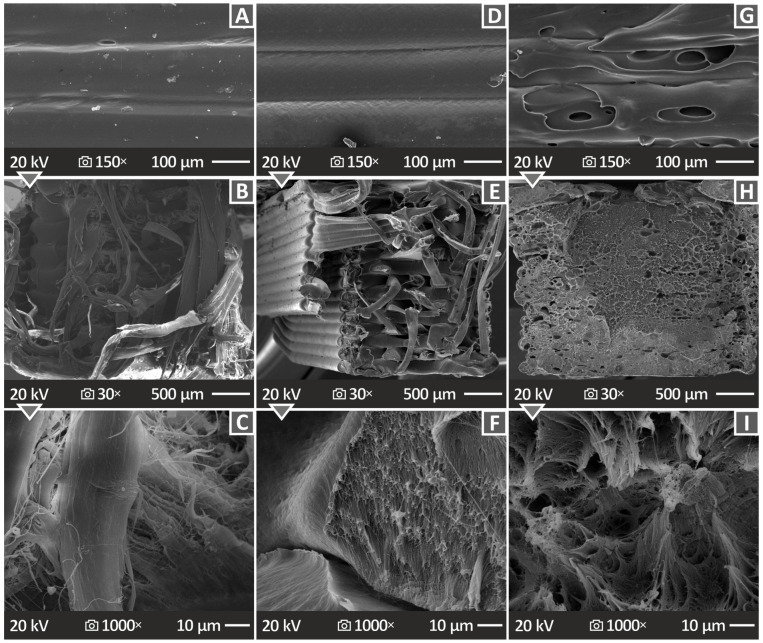
SEM images of (**A**–**C**) pure HDPE side surface at 150× magnification, fracture surface at 30× magnification, and fracture surface at 1000× magnification, respectively, (**D**–**F**) HDPE/10 wt.% CB side surface at 150× magnification, fracture surface at 30× magnification, and fracture surface at 1000× magnification, respectively, and (**G**–**I**) HDPE/20 wt.% CB side surface at 150× magnification, fracture surface at 30× magnification, and fracture surface at 1000× magnification, respectively.

The microstructure and morphology of the specimens were investigated by analyzing the captured SEM images of both the samples’ sides and fracture surfaces. Figure 11 presents SEM images of the pure HDPE, HDPE/10 wt.% CB, and HDPE/20 wt.% CB at various magnifications. Figure 11A–C show SEM images of pure HDPE, namely, the side surface at 150× magnification and fracture surface at 30× and 1000× magnifications, respectively. Figure 11D–F show SEM images of the HDPE/10 wt.% CB, namely, the side surface at 150× magnification and the fracture surface at 30× and 1000× magnifications, while Figure 11G–I show SEM images of the HDPE/20 wt.% CB, namely, 150× magnification of the side surface and 30× and 1000× magnifications of the fracture surface, respectively. Figure 11A,D show a well-distributed layering, unlike the layering presented in Figure 11G, which is uneven and contains pores and voids. The specimens of Figure 11B,E, in low magnification (30×), present protrusive filament in the fracture surface, and the surfaces are rough, while the specimen in Figure 11H is less rough and more solid, without filament protrusion. The pure HDPE presents high deformation in the higher magnification image (Figure 11C), while the HDPE/10 wt.% CB (Figure 11F) and HDPE/20 wt.% CB (Figure 11C) at 1000× magnification present a ductile fracture surface.

**Figure 12:**
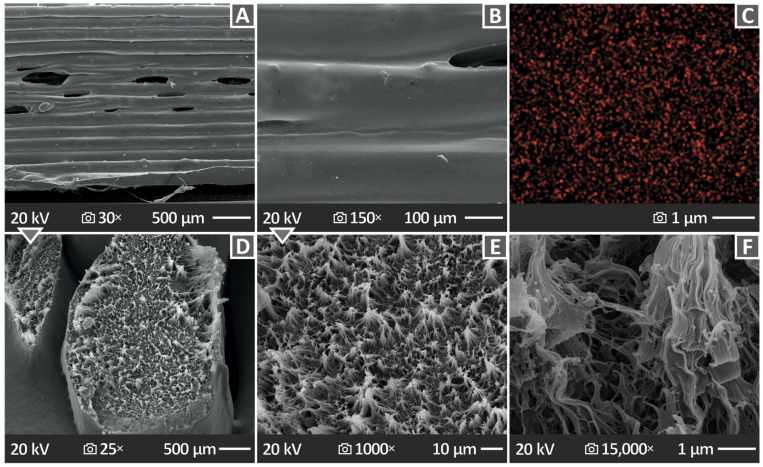
(**A**,**B**) SEM images of HDPE/16.0 wt.% CB side surface at 30× and 150× magnification and, correspondingly, (**C**) a MAP image of HDPE/16.0 wt.% CB, (**D**–**F**) SEM images of HDPE/16.0 wt.% CB fracture surface at 25×, 1000×, and 15,000× magnifications, respectively.

Figure 12 shows the side and fracture surface SEM images of the HDPE/16.0 wt.% CB along with an EDS mapping image of the carbon element. Figure 12A,B present the side surface at 30× and 150× magnification, respectively. Figure 12C is the MAP image, and Figure 12D–F present the fracture surface of the HDPE/16.0 wt.% CB at 25×, 1000×, and 15,000× magnifications. The side surface images present some voids between the layers, while the fracture surface images show a less ductile form, in relation to those of the pure HDPE, HDPE/10 wt.% CB, and HDPE/20 wt.% CB in Figure 11.

## 4. Discussion

In this study, CB was used as a filler for HDPE in eight different filler concentrations (2.0, 4.0, 6.0, 8.0, 10.0, 16.0, 20.0, and 24.0 wt.%) in order to produce filaments and by extension specimens, to be examined through a variety of tests and compared with the performance of pure HDPE. The tensile and flexural results showed great performance, in comparison to pure HDPE, in the case of the HDPE/20.0 wt.% CB, as they were all improved by 53.0% or more. Moreover, Charpy the impact strength of the HDPE/2.0 wt.% CB was increased by 29.9% above pure HDPE, and the microhardness of the HDPE/24.0 wt.% CB also showed an increase of 9.1% above pure HDPE. As to the electrical conductivity, it increased as the filler percentage rose, and the highest value was found in the case of the HDPE/24.0 wt.% CB, with the 20 wt.% loaded composites, which showed the highest improvement in mechanical performance overall, having very similar values.

By observing the side surface SEM images of the fabricated specimens, it was indicated that the layering was mostly well-distributed, but also porous in some cases, while the fracture surface images showed a ductile form. The pores observed in the higher-loaded composites indicate that the 3D printing settings were not the most appropriate for these composites. The reduction in the MFR with the increase in the CB loading in the composites supports the argument that the 3D printing settings needed better adjustment of each composite. As mentioned, the 3D printing settings were optimized for pure HDPE and used in all the materials, for comparison purposes. So, the reduction in the MFR instructs for different 3D printing settings in the nanocomposites to achieve optimum results, which is the desired goal in actual 3D printing applications. The MFR, as shown in Figure 6, gradually decreased with the increase in the filler content. So, for each nanocomposite, the optimum settings should be found. With the settings used in the research, it was possible to 3D print the samples, and no serious processability issues, such as nozzle clogging, or others, occurred during the process even for the high-loaded nanocomposites. By the experimental results, the 3D-printed samples achieved improved performance in the tests, up to a specific filler loading, in which saturation of the filler in the matrix started to occur. So, the results can be considered as acceptable as in the study. Still, since the MFR decreased, the 3D printing temperature increase can compensate for this decrease, while other parameters, such as the 3D printing speed, might need adjustment as well to achieve the best possible fusion in the samples, in all nanocomposites, especially the highly loaded ones.

On the other hand, as mentioned, the temperatures used did not cause any degradation in the composites, which would have affected the mechanical test results. Additionally, the thermal stability of the HDPE polymer was not significantly affected by the addition of the CB particles. A similar behavior is reported in the literature by the addition of titania in the HDPE polymer, with the composites prepared for the MEX 3D printing method as well, with a similar method as the one followed herein [3].

By the carried-out tests and measurements, it is safe to assume that, with the process followed, a sufficient dispersion of the particles in the matrix was achieved. The data that support this argument are related to (a) the two-step extrusion process followed, (b) the specially designed extruder in the second step for material mixing, (c) the lack of particle clustering in the SEM images of the fracture areas, (d) which was verified also in the EDS mapping for the carbon element, in which a uniform distribution in the observation regions is depicted in the images, and finally (e) the acceptable deviation in the mechanical tests, which indicates a similar materials composition in each sample (five were tested per experiment and composite).

By the literature review, according to the authors’ best knowledge, no similar composites have been presented in the literature so far in MEX 3D printing. Still, research has investigated HDPE/CB composites, but not for their mechanical performance, with composites prepared with a different method than the one presented herein. In [56], HDPE combined with CB was used to investigate how heat treatment influenced the composite’s electrically conductive behavior. It was concluded that the annealing temperature had a great impact on both the resistivity and the positive temperature coefficient (PTC) intensity of HDPE/CB composites. In an additional study, various filler percentages of CB added to HDPE have been investigated. The filler concentrations were 15.0, 19.0, and 25.0%, close to those investigated in the study herein [76].

The [77] investigation examined PA12/CB composites of 0.1, 0.5, 1.0, 2.5, 5.0, and 10.0 wt.% filler concentration, which was utilized for the fabrication of 3D-printed samples. The investigation included the observation of the samples’ mechanical performance and electrical percolation as well as an analysis of their fracture mechanism through SEM images, similar to the study herein. The most remarkable reinforcement was observed in the case of the PA12/5.0 wt.% CB, while the electrical conductivity was exhibited from the 2.5 wt.% filler percentage and above. Moreover, it should be noted that, by increasing the quantity of filler, the electrical conductivity was also increased, as also happened in the HDPE/CB samples of this study.

An investigation has been completed on PP/CB composites suitable for 3D printing but did not focus on the mechanical properties as the study herein does [78]. CB and Ag microscale particles have been used as fillers, with up to 0.75 wt.% concentration, for PET in order to fabricate samples by hot pressing, where it was observed that tensile strength was increased in the case of 0.5 wt.% [51]. By the studies discussed above, it is shown that CB is mainly used for its electrical properties for the development of composites, with some works presented for 3D printing applications, but with different matrices. Still, no study so far has reported such an impressive improvement in the mechanical performance of polymeric matrices, as the current study does, showing the potential of CB as a reinforcement agent as well.

## 5. Conclusions

Herein, composite filaments compatible with the MEX 3D printing process were fabricated out of various percentages of the CB filler up to 24 wt.%, using HDPE as the matrix material. HDPE was selected due to its popularity in various applications in packaging and films, especially when chemical resistance is required. CB was selected as the filler to investigate the possibility of inducing electrical properties in the HDPE matrix, apart from the mechanical reinforcement. The electrical properties were confirmed in the study, and the mechanical properties of the HDPE matrix were also notably improved.

**Figure 13:**
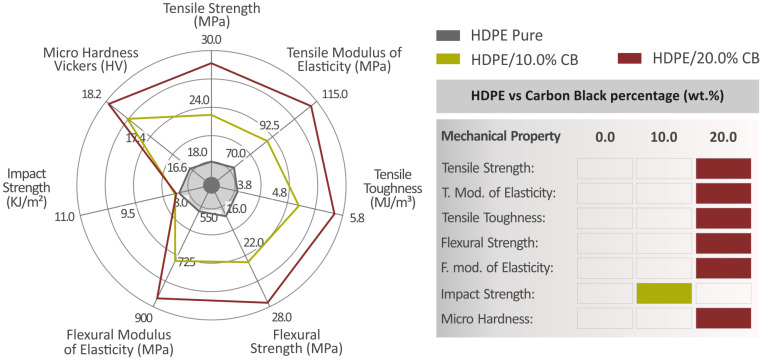
Summary of the results, regarding the mechanical properties of pure HDPE, HDPE/10.0 wt.% CB, and HDPE/20.0 wt.% CB (left side of the figure), and the presentation of the highest values, regarding all the mechanical properties of pure HDPE, HDPE/10.0 wt.% CB, and HDPE/20.0 wt.% CB (right side of the figure).

The filaments were utilized to manufacture respective specimens through material extrusion 3D printing. Specimens were made to be suitable for a range of standard tests to investigate their mechanical and thermomechanical properties. Raman spectroscopy, TGA, DSC, Rheology, and MFR analyses were employed, while tensile, flexural, and impact tests were carried out. The results regarding the tensile strength, tensile modulus of elasticity, tensile toughness, flexural strength, flexural modulus of elasticity, impact strength, and microhardness of pure HDPE, HDPE/10.0 wt.% CB, and HDPE/20.0 wt.% CB are summarized and presented on the left side of Figure 13, along with a table that indicates the composite that presents the highest value of each mechanical property on the right side of Figure 13. The HDPE/20.0 wt.% CB presented the highest values of all the mechanical properties except for the impact strength where HDPE/10.0 wt.% CB excelled. It can be concluded that the filler of CB worked as a great reinforcement to HDPE in order to optimize its mechanical performance. The HDPE/20.0 wt.% CB showed an impressive 57.8% improvement in the tensile strength, compared to pure HDPE, 54.4% in the tensile modulus of elasticity, 59.7% in the flexural strength, and 53% in the flexural modulus of elasticity. At the same time, this composite (HDPE/20.0 wt.% CB) showed the second-highest electrical conductivity (but very close to the highest one), which is an additional merit for the composites investigated. Such composites can be exploited in applications satisfying respective requirements, which is an asset not often found in MEX 3D printing materials. In future work, additional tests and further characterization techniques can be carried out. The process can be adjusted for industrial use, and the 3D printing settings can be optimized for the composites, further improving their performance. This was not completed herein, as the same settings were used in all composites and pure HDPE to have comparable results.

## Figures and Tables

**Table 1:** Significant Raman peaks and their related assignments from pure HDPE.

**Wavenumber** (**cm^−1^**)	**Intensity**	**Raman Peak Assignment**
1064	Medium	C-O-C stretching [70]
1131	Medium	C-O-C stretching [71]
1297	Medium	C-O-C stretching [70]
1418	Small	CH_3_ deformation [70]
1441	Small	CH_2_ deformation [70,72]
2850	Major	CH_2_ symmetric stretching [73]
2884	Major	C-H antisymmetric stretching [74]

## Data Availability

Data are contained within the article.
